# Supplementary material for: Effect of Nanoemulsion Containing Enterocin GR17 and Cinnamaldehyde on Microbiological, Physicochemical and Sensory Properties and Shelf Life of Liquid-Smoked Salmon Fillets
Source: Foods. 2022 Dec 23;12(1):78. doi: 10.3390/foods12010078 (PMC9818589; doi:10.3390/foods12010078)
Supplement: Supplementary file 1 [file foods-12-00078-s001.zip › foods-2070164-SI.pdf]

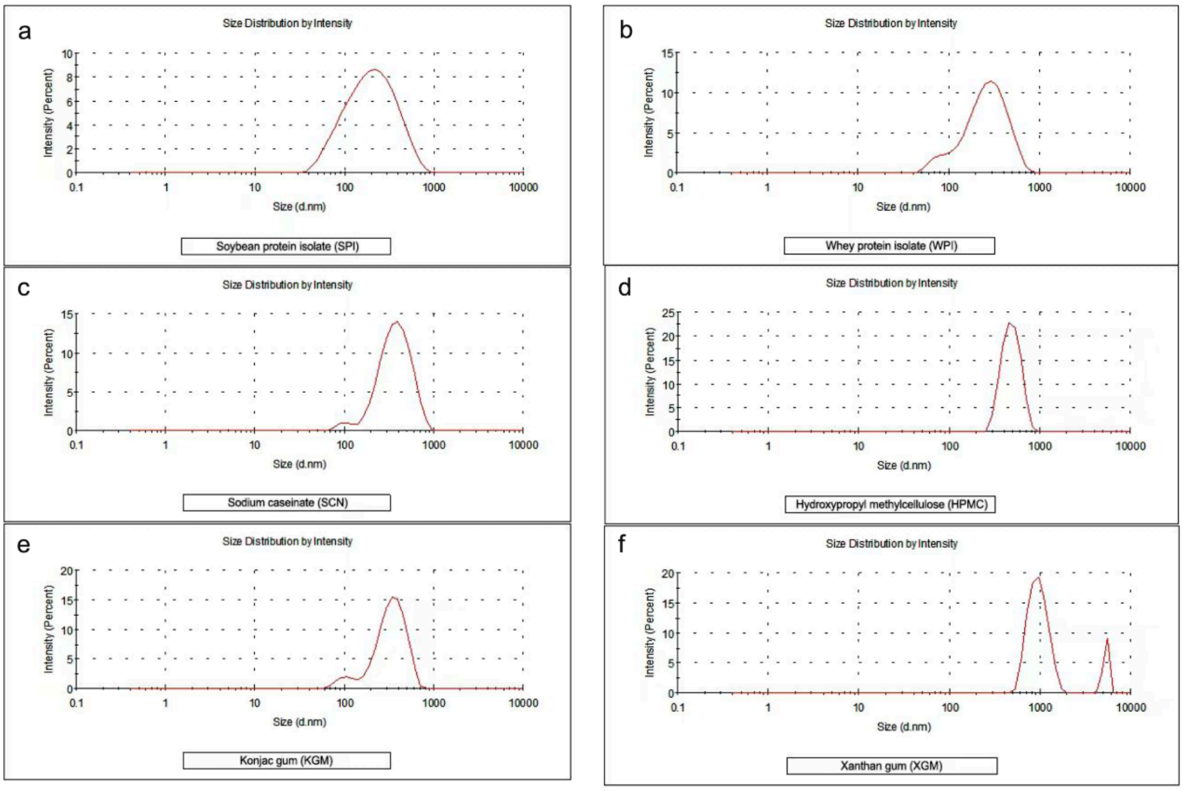

Figure S1. The droplet size of nanoemulsions containing enterocin Gr17 and cinnamaldehyde produced by using soybean protein isolate (SPI), whey protein isolate (WPI), sodium caseinate (SCN), hydroxypropyl methyl-cellulose (HPMC), konjac gum (KGM) and xanthan gum (XGM).

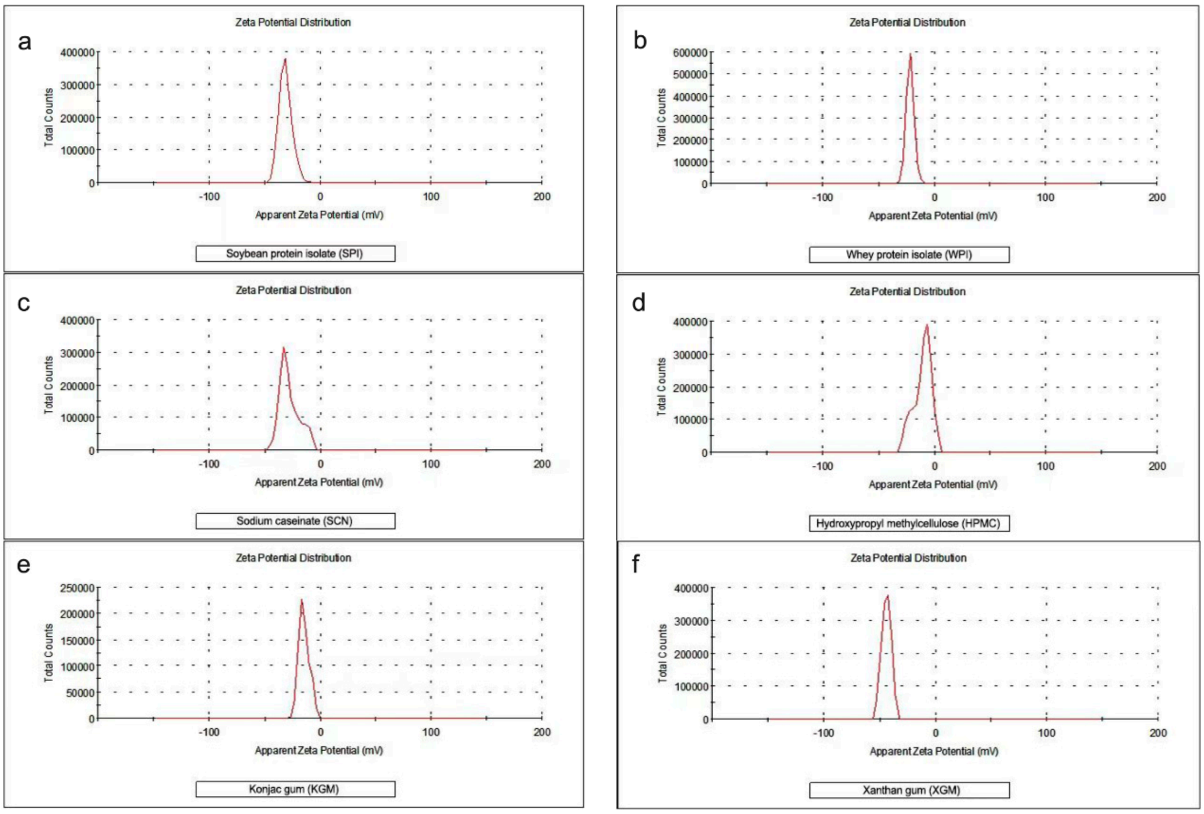

Figure S2. The zeta potential of nanoemulsions containing enterocin Gr17 and cinnamaldehyde produced by using soybean protein isolate (SPI), whey protein isolate (WPI), sodium caseinate (SCN), hydroxypropyl methyl-cellulose (HPMC), konjac gum (KGM) and xanthan gum (XGM).

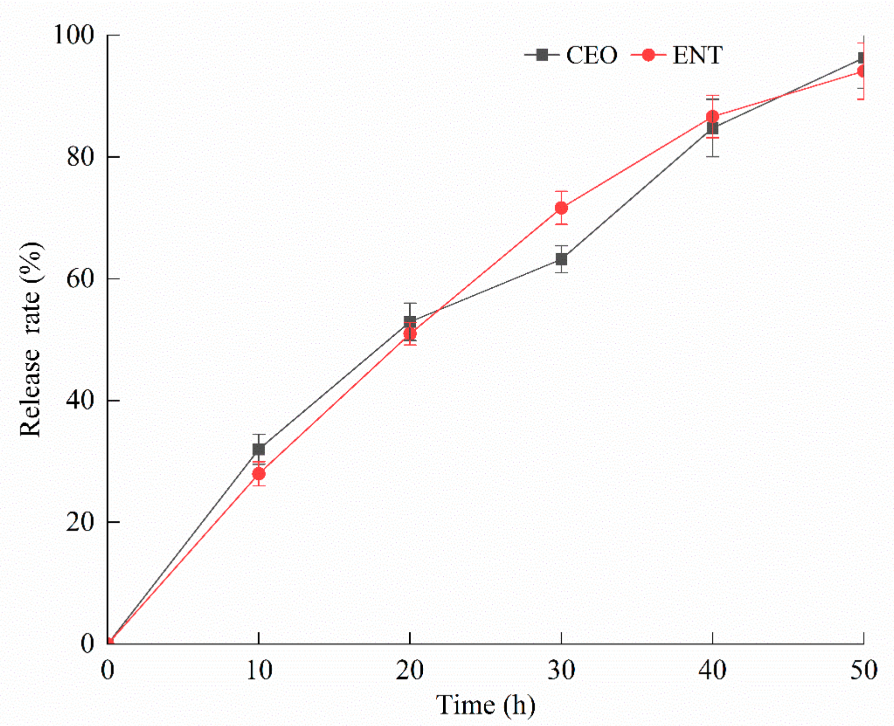

Figure S3. The release rate of enterocin Gr17 (ENT)/cinnamaldehyde (CEO) in nanoemulsion produced by using soybean protein isolate (SPI).

Table S1. Identification of the volatile compounds of smoked salmon fillets

Compounds	RI	Thresholds (μg/L)	Odor description
Aldehydes			
3-methyl-Butanal	643	1.1	Fatty, chocolate, peachy
Hexanal	806	5	Fishy, grassy, aliphatic aldehyde-acid
Heptanal	905	2.8	Fruity, grassy
Benzaldehyde	1434	750.89	Bitter almond
Octanal	1005	0.587	Waxy, citrus peel
Nonanal	1104	1.1	Fatty, waxy, rose, citrusy
Bnzeneacetaldehyde	1529	6.3	Hyacinth, chocolate
Decanal	1204	3	Citrus peel
Undecanal	1065	12.5	Floral, rose, sweet orange
Alcohols			
1-Penten-3-ol	1109	358.1	-
1-Hexanol	1287	5.6	Fruity
1-Pentanol	1193	150.2	Special smell
1-Octen-3-ol	1109	1.5	Mushroom, earthy, fatty, raw chicken
3-methyl-Butanol	1153	4	Fusel oil, alcohol, banana
Phenylethylalcohol	1787	564.23	Rose
Ketones			
2-methyl-3-Pentanone	1209	80	-
2,3-Pentadione	1068	40	-
2-Nonanone	1316	82	-
Esters			
Ethyl acetate	586	5	Sweet orange ether, floral, bouquet
Isoamyl formate	1618	149	Fruity
Methylsalicylate	1665	40	Wintergreen oil, minty
n-Butylbutanoate	1743	400	Fruity
2,2,4-Trimethyl-1,3-pentanediol diisobutyrate	1605	0.014	-
Diethyl Phthalate	1639	3.3	-
Acids			
Acetic acid	2073	9.9	Pungent
Amines			
Dimethylamine	1056	30000	Stench
Trimethylamine	1047	23	Fishy, oil stink, hala flavor、Stale sweat

Hydrocarbons			
1-Pentene	508	0.29	Putrid odor
n-Hexane	618	5.3	Petroleum
Hexadecane	1612	500	-
Octadecane	0.02	1810	
Aromatic compounds			
Toluene	794	527	Pungent
p-Xylene	907	1000	Toluene
2-Amylfuran	1172	5.8	Bean、fruity、earthy、Green、vegetable fragrance
2-methoxy-Phenol	1090	1.6	Woody、smoky、spice、Vanilla、meaty
Creosol	1203	30	Vanilla bean, picees
Thymol	1262	1700	Spicy
2-(1,1-dimethylethyl)-4-methyl-Phenol	1341	30	Leather
Sulfur compounds			
Carbondisulfide	-	5	Fragrance, rotten radish
